# *CTBP1* and *CTBP2* mutations underpinning neurological disorders: a systematic review

**DOI:** 10.1007/s10048-022-00700-w

**Published:** 2022-11-04

**Authors:** Natalia Acosta-Baena, Johanna Alexandra Tejada-Moreno, Mauricio Arcos-Burgos, Carlos Andrés Villegas-Lanau

**Affiliations:** 1 Grupo de Genética Molecular (GENMOL), Universidad de Antioquia, Medellín, Colombia; 2 Grupo de Neurociencias de Antioquia (GNA), Facultad de Medicina, Universidad de Antioquia, Medellín, Colombia; 3 Grupo de Investigación en Psiquiatría (GIPSI), Departamento de Psiquiatría, Instituto de Investigaciones Médicas, Facultad de Medicina, Universidad de Antioquia, Medellín, Colombia

**Keywords:** Transcriptional corepressors, CTBP, Neurodevelopment, HADDTS syndrome, De novo mutations, R342W, Recurrent mutation, PLDLS motif

## Abstract

C-terminal binding proteins (CtBP1/2) are transcriptional coregulators that play a significant role during vertebrate neurodevelopment. This systematic review aims to identify case reports with genetic variants in *CTBP1* and *CTBP2* associated with brain development syndromes.

We screened different databases (PubMed, Scopus, Google Scholar, LILACS) by systematically searching journals and checking reference lists and citations of background papers. We found fourteen cases (10 males) from five papers carrying two pathogenic, heterozygous variants in the *CTBP1* gene (13 individuals carried the missense mutation c.991C T, p.Arg342Trp, and one subject carrying the 2-base pair deletion c.1315_1316delCA, p.Gln439ValfsTer84). These mutations were de novo in 13 cases and one case of maternal germinal mosaicism. Two variants are in the same domain of the protein: Pro-Leu-Asp-Leu-Ser (PLDLS) C terminal. Patients with these mutations exhibit a phenotype with intellectual disability, HADDTS syndrome (hypotonia, ataxia, developmental delay, and tooth enamel defects), and cerebellar volume loss. We did not identify reported cases associated with homozygous mutations harbored in CTBP1. We did not identify any report of neurodevelopment phenotypes associated with heterozygous or homozygous *CTBP2* mutations. Due to CTBP2/RIBEYE being a gene with dual function, identifying and interpreting the potential pathogenic variants is challenging.

Further, homozygous mutations in the CTBP2 gene may be lethal. The mechanisms involved in the pathogenesis of neurodevelopment due to variants of these proteins have not yet been elucidated, despite some functional evidence. Further studies should be conducted to understand these transcription factors and their interaction with each other and their partners.

## Introduction

C-terminal binding proteins (*CTBP1* and *CTBP2*) are two highly conserved proteins expressed in different tissues of vertebrate species [3] and share 76% homology [4]. The primary function of the *CTBP* family members is to be a transcriptional corepressors. Since these proteins do not bind directly to DNA, they form a corepressor complex to perform their function by developing dimers with chromatin-modifying enzymes (histone deacetylases and methyltransferases), DNA-binding proteins, chromodomain-containing proteins, and CoREST proteins [5]. Other functions are controlling the equilibrium between tubular and stacked structures in the Golgi complex and brown adipose tissue differentiation.

*CTBPs* have three main domains: The substrate-binding domains, which contain the Pro-X-Asp-Leu-Ser (PXDLS) binding sequence, the central domain Arg-Arg-Thr (RRT), responsible for NAD(H) binding and dimerization, and a C-terminal domain. The partners of *CTBPs* are sequence-specific that bind to the PXDLS domain [2]. Though *CTBP 1/2* share similar functions, they have some differences. The *CTBP1* gene is on chromosome 4p, and *CTBP2* is on chromosome 10q. Both proteins are ubiquitously expressed in all human tissues. However, *CTBP2* appears to be expressed earlier in development. Only CTBP2 has a nuclear localization signal at its N-terminus. Conversely, *CTBP1* has a PDZ-binding domain at its C-terminus for cytoplasmic functions with particular proteins such as neuronal nitric oxide synthase [6]. *CTBPs* have alternative splicing. *CTBP2* has two dual functions with each type of isoform. The *CTBP2* isoform has the function of a transcription factor. The isoform called *RIBEYE* is the main component of synaptic ribbons or specialized synapses. CTBPs can form homodimers or heterodimers necessary to carry out their functions [2], but this relevance is not fully known.

CTBP family members have been implicated in critical functions for neural development in various species, including drosophila, xenopus [4], mice [7], and avians [6]. *CTBPs* have been implicated in developing neural tube closure, forebrain, and hindbrain in murine [6, 7]. In humans, although *CTBPs* have precise functions in brain development, few studies have focused on the exact role. Most studies are focused on cancer due to the participation of these transcription factors in various functions associated with cell proliferation and apoptosis. With this review, we want to identify possible polymorphisms in *CTBP 1/2* that have been associated with or suggested as gene candidates for phenotypes in the human nervous system.

## Methods

### Key question

Have cases been reported with genetic variants in *CTBP1* or *CTBP2* genes associated with neurological, neurodegenerative, or neurodevelopmental diseases?

### Eligibility criteria

- Types of studies: case reports and case series were included. No language, publication date, or publication status restrictions were imposed.
- Types of participants: humans. No restriction by mode of inheritance or transmission, nor by the type of variant or classification.
- Types of comparison/intervention: genetic variants (exon or intron) in C-terminal binding proteins (*CTBP1/2*), without sequencing or genetic analysis restriction.
- *Types of outcome measures:* all reports of clinical cases diagnosed with neurological, neurodegenerative, or neurodevelopmental diseases, including neural tube defects.

### Information sources and selection

Studies were identified by searching electronic databases: PubMed, Scopus, Google Scholar, and LILACS. Other sources were hand searching of genetic journals, preprint server Health Science Case Reports Research Network (https://www.ssrn.com/index.cfm/en/hscasereprn/); DECIPHER database (https://www.deciphergenomics.org/), checking reference lists and citations of background papers. The search end date was 09 Jun 2022.

### Search methods for the identification of studies

The following search strategies were used:

1. MEDLINE—PubMed
  The PubMed search strategy used is available in Table 1. We used the following search terms: “nervous system development,” nervous system embryology,” “neurodevelopmental disorders,” “intellectual disability,” “neural tube defect,” “CTBP2,” “CTBP1,” “humans,” “RIBEYE,” “BARS protein,” “C-terminal Binding Protein,” “Brefeldin A-Ribosylated Substrate,” “case series study,” “genetic association studies,” and “case report.”

**Table 1:** PubMed search strategy

Search	Query	Results
#1	Search: CTBP1	321
#2	Search: CTBP2	345
#3	Search: RIBEYE	427
#4	Search: BARS protein	915
#5	Search: C-Terminal Binding Protein	651
#6	Search: Brefeldin A-Ribosylated Substrate	3
#7	Search: (((((CTBP1) OR (CTBP2)) OR (RIBEYE)) OR (BARS protein)) OR (C-Terminal Binding Protein)) OR (Brefeldin A-Ribosylated Substrate)	2213
#8	Search: neurodevelopmental disorders	212,788
#9	Search: intellectual disability	112,546
#10	Search: central nervous system embryology	63,019
#11	Search: nervous system development	420,675
#12	Search: nervous system embryology	85,032
#13	Search: neural tube defect	33,619
#14	Search: (((((neurodevelopmental disorders) OR (intellectual disability)) OR (central nervous system embryology)) OR (nervous system development)) OR (nervous system embryology)) OR (neural tube defect)	730,858
#15	Search: humans	21,317,467
#16	Search: a case report	2,331,614
#17	Search: genetic association studies	152,52
#18	Search: case series study	105,312
#19	Search: (((case series study)) OR (genetic association studies)) OR (case report)	2,568,788
#20	Search: ((humans) AND (((((((neurodevelopmental disorders) OR (intellectual disability)) OR (central nervous system embryology)) OR (nervous system development)) OR (nervous system embryology)) OR (neural tube defect)) AND ((((((CTBP1) OR (CTBP2)) OR (RIBEYE)) OR (BARS protein)) OR (C-Terminal Binding Protein)) OR (Brefeldin A-Ribosylated Substrate)))) AND ((((case series study)) OR (genetic association studies)) OR (case report))	7
  The final searches were ((humans) AND (((((((neurodevelopmental disorders) OR (intellectual disability)) OR (central nervous system embryology)) OR (nervous system development)) OR (nervous system embryology)) OR (neural tube defect)) AND ((((((CTBP1) OR (CTBP2)) OR (RIBEYE)) OR (BARS protein)) OR (C-Terminal Binding Protein)) OR (Brefeldin A-Ribosylated Substrate)))) AND ((((case series study)) OR (genetic association studies)) OR (case report)).
2. SCOPUS
  The search was carried out in documents by keyword/title or abstract without any restriction or filter (Table 2). We used the following search terms: “nervous system development,” “nervous system embryology,” “neurodevelopmental disorders,” “intellectual disability,” “neural tube defect,” “CTBP,” and “C-Terminal Binding Protein.”

**Table 2:** Scopus search strategy

Search	Query	Results
5	( ( TITLE-ABS-KEY ( ctbp)) OR ( TITLE-ABS-KEY ( "c-terminal binding protein"))) AND ( ( TITLE-ABS-KEY ( neurodevelopmental AND disorders) OR TITLE-ABS-KEY ( nervous AND system AND embryology) OR TITLE-ABS-KEY ( nervous AND system AND development) OR TITLE-ABS-KEY ( intellectual AND disability) OR TITLE-ABS-KEY ( neural AND tube AND defect)))	21
4	( TITLE-ABS-KEY ( neurodevelopmental AND disorders) OR TITLE-ABS-KEY ( nervous AND system AND embryology) OR TITLE-ABS-KEY ( nervous AND system AND development) OR TITLE-ABS-KEY ( intellectual AND disability) OR TITLE-ABS-KEY ( neural AND tube AND defect))	264,152
3	( TITLE-ABS-KEY ( CTBP)) OR ( TITLE-ABS-KEY ( "c-terminal binding protein"))	961
2	C-Terminal Binding Protein	
	TITLE-ABS-KEY ( "c-terminal binding protein")	703
1	CTBP	
	TITLE-ABS-KEY ( CTBP)	598
  The final searches were ((TITLE-ABS-KEY (CTBP)) OR (TITLE-ABS-KEY (“C-Terminal Binding Protein”))) AND ((TITLE-ABS-KEY (neurodevelopmental AND disorders) OR TITLE-ABS-KEY (nervous AND system AND embryology) OR TITLE-ABS-KEY (nervous AND system AND development) OR TITLE-ABS-KEY (intellectual AND disability) OR TITLE-ABS-KEY (neural AND tube AND defect))).
3. LILACS (Latin American and Caribbean Health Sciences database)
  The search was carried out in subject/title/abstract. The term used was “CTPB.”
4. Google Scholar
  We used the same search terms used in PubMed combined with Boolean connectors.

### Data extraction and analysis

The title and the abstract initially selected the articles returned by the searches. We read the full text of pre-selected studies and included papers that met the above criteria. Finally, the articles selected for the review were checked to avoid duplicate published data.

## Results

### Selection of studies

**Fig. 1:**
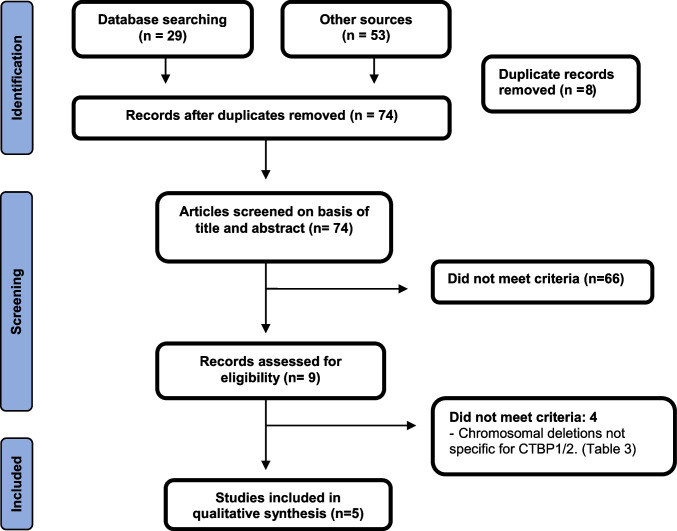
Flow diagram of study selection, following the PRISMA guidelines [8]

The search carried out to select the studies included in this review is detailed in Fig. 1. A total of 78 references were identified, with potentially valuable articles in PubMed = 7, Scopus = 21, and LILACS = 1. Google Scholar and hand searching were found an additional 49 studies. After adjusting for duplicate studies, 74 studies remained, which were screened by title and abstract. Of these, reports that did not meet the inclusion criteria were excluded, leaving us to review nine articles in full. In the analysis, five studies that met the inclusion criteria were included.

In DECIPHER database, a missense variant in CTBP2 is reported (c.979G > C, p.Glu327Gln), associated with an autism spectrum phenotype, cleft palate, diffuse white matter abnormalities, and severe intellectual disability. The variant was de novo and heterozygous. There is no published paper confirming the variant. In addition, the genotype of the reported individual appears associated with other additional variants in AUTS2 (c.3566 T > C, p.Leu1189Pro) and ITGB3 (c.985A > G, p.Asn329Asp) [9].

### Characteristics of included studies

In our review, a total of 9 studies were identified in which a member of the CTBP family was involved. Within this search, there were studies reporting cases with distal chromosomal deletions on chromosomes 4p and 10q, where the syndrome was not specific for *CTBP1* and *CTBP2*, respectively. Therefore, they were excluded from the phenotype analyses. Five included studies were summarized as shown in Table 3. Four excluded studies were summarized in Table 4.

**Table 3:** Five studies included in the analysis

Study	Methods	# cases reported	Gene	Variant/type/allele	Position Gen/protein	inheritance	Sanger confirmation	Functional studies
Beck et al. [10]	Whole exome sequencing (WES)	5 cases (4 affected/one healthy)	***CTBP1***	c.991CT, p.Arg342Trp Missense Heterozygous	Exon 8/PLDLS binding cleft domain	De novo = 3 Somatic mosaicism = 2	Yes	No (functional studies of these same cases were reported in study #3)
Sommerville et al. [11]	WES mitochondrial genome sequencing	1	***CTBP1***	c.991CT, p.Arg342Trp Missense Heterozygous	Exon 8/PLDLS binding cleft domain	De novo	Yes	Decreased respiratory chain activities of complexes I and IV
Beck et al. [12]	WES	7	***CTBP1***	c.991CT, p.Arg342Trp Missense Heterozygous	Exon 8/PLDLS binding cleft domain	De novo	Not mentioned in this report	-Proteomic analysis: reduced interaction of chromatin-modifying factors with the CtBP1 mutant -Genome-wide transcriptome analysis in human glioblastoma cell lines expressing the CtBP1 mutation: Showed changes in the expression profiles of genes that control multiple cellular processes -Patient-derived dermal fibroblasts were more sensitive to apoptosis during glucose deprivation
Bhatia et al. [13]	WES	1	***CTBP1***	c.991CT, p.Arg342Trp Missense Heterozygous	Exon 8/PLDLS binding cleft	De novo	Yes	No
Khamirani et al. [14]	WES	1	***CTBP1***	c.1315_1316delCA, p.Gln439ValfsTer84 2-pb deletion Heterozygous	Not analyzed	Consanguineous parents. A proband with an affected brother with a similar clinical condition (not studied)	Yes	No

**Table 4:** Excluded studies report cases with distal chromosomal deletions on chromosomes 4p and 10q

Study	Associated syndrome	Methods	# cases reported	Gene	Variant/type/allele	Position Gen/protein	Inheritance	Sanger confirmation	Functional studies
Shimizu et al. [15]	Wolf–Hirschhorn syndrome (WHS)	G-banded karyotyping Fluorescence in situ hybridization (FISH) Chromosomal microarray analysis (whole-genome oligonucleotide-based array platforms)	21 (one individual of the 22 reported does not include a deletion in the CTBP1 gene)	***CTBP1*** (4p16.3) among others	Distal chromosome 4p deletion	Chromosome 4p hemizygosity of CTBP1	De novo = 20 Maternal = 1	Not apply	No
Callaway et al. [16]	Wolf–Hirschhorn syndrome (WHS)	Chromosomal microarray analysis	1	***CTBP1*** (4p16.3) among others	Distal chromosome 4p deletion	Chromosome 4p hemizygosity of CTBP1	De novo	Not apply	No
Irving et al. [17]	Partial deletion of the long arm of chromosome 10	Karyotyping FISH	1 case with breakpoint 26.13–26.3 0f 15 reported with Chr.10q deletions	***CTBP2*** (10q26.13) among others	Distal chromosome 10q deletion: del(10)(q26.13–26.3)	Chromosome 10q hemizygosity of CTBP2	De novo	Not apply	No
Vera-Carbonell et al. [18]	The 10q26 deletion syndrome	Karyotyping FISH Oligo array-CGH analysis	3	***CTBP2*** (10q26.13) among others	Distal chromosome 10q26 deletion	Chromosome 10q hemizygosity of CTBP2	De novo	Not apply	No

#### Study design

We identify three case reports [11, 13, 14] and two case series reports [10, 12]. All five studies identified variants by whole-exome sequencing (WES). Sanger confirmed four reports and two studies with additional functional studies [11, 12].

#### Identified variants

Two variants have only been reported in the CTBP1 gene. A variant (c.1315_ 1316delCA, p.Gln439ValfsTer84) has been reported in a single case, confirmed by Sanger but without functional studies [14]. The other 13 cases present the same recurrent heterozygous mutation (c.991CT, p.Arg342Trp). Beck et al. [10] report that case 1 presents another addition variant (in COL6A3) to CTBP1 with maternal somatic mosaicism. This study also reports that the mother of this same individual is healthy despite having this mosaicism.

#### Description of the cases

Fourteen individual cases and clinical characteristics were summarized in Tables 5 and 6. The nationality of the cases is not recorded in the publications. Eleven cases were described in the USA (cases 1–11, Table 4). One case was reported in the UK (case 12, Table 4), another in Iran (case 13, Table 4), and the last in India (case 14, Table 4). Severe intellectual disability (ID) or global development delay was present in twelve cases—eleven cases with significant gait disturbance, including 3 cases without gait. Cerebellar atrophy was identified in nine subjects. None of the cases reported seizures, except case 14, with a history of a single episode of myoclonus at 5 years of age. Three cases did not report defects in dental enamel.

**Table 5:** The main features of the 14 identified cases with CTBP1 variants

Case	Age/gender	Facies/general characteristics	Intellectual disability/global development delay	Oculomotor apraxia	Gait disturbance	Developmental regression	Reference
1	8 years/M	-	Borderline normal	Not described	+	-	Beck et al. [10]
2	20 years/M	Frontal bossing, deep-set eyes	+ Severe	+	+ Nonambulatory	-	Beck et al. [10]
3	9 years/F	Retrognathia highly arched palate	-	+	+ Nonambulatory	-	Beck et al. [10]
4	12 years/F	-	+ Severe	+	+	-	Beck et al. [10]
5	20 years/M	Not described	+	Not described	+	-	Beck et al. [12]
6	22 years/F	Not described	+	+	+ wide-based gait, requiring support to take steps	+	Beck et al. [12]
7	6 years/M	Not described	+	Not described	Not described	-	Beck et al. [12]
8	6 years/M	Not described	+	Not described	Not described	-	Beck et al. [12]
9	10 years/M	Not described	+	Not described	+	+ Motor, cognitive	Beck et al. [12]
10	5 years/M	Not described	+	-	Not described	+ Motor	Beck et al. [12]
11	11 years/M	Not described	+	+	+ wide-based gait Required full support to stand and walk	-	Beck et al. [12]
12	16 years/F	Sunken eyes and thin tapering fingers	+	-	+ Nonambulatory She used a wheelchair	+ Motor, language	Sommerville et al. [11]
13	7 years/M	Long face, the teeth were irregular, widely spaced upper incisors	+	Not described	+ loss of ambulation at around 5 years of age	Not described	Bhatia et al. [13]
14	25 years/M	Not described	+	-	Slightly wide-based gait and difficulty with balance	-	Khamirani et al. [14]

**Table 6:** The main features of the 14 identified cases with CTBP1 variants

Case	Dysarthria	Muscle weakness	HADDTS syndrome (hypotonia, ataxia, developmental delay, and tooth enamel defects)	Cerebellar atrophy	Reference
1	+	+	+	+	Beck et al. [10]
2	+	+	+	-	Beck et al. [10]
3	+	+	+	+	Beck et al. [10]
4	+	+	+	-	Beck et al. [10]
5	-	-	Not described tooth enamel defects	Not described	Beck et al. [12]
6	+	+	+	+ Cerebellar and cerebral atrophy	Beck et al. [12]
7	Not described	Not described	+	-	Beck et al. [12]
8	Not described	Not described	Ataxia not described	+	Beck et al. [12]
9	Not described	Not described	+ Axial hypotonia	+ Mild Dandy-Walker cyst	Beck et al. [12]
10	+	Not described	Not described tooth enamel defects	+ Cerebellum was underdeveloped	Beck et al. [12]
11	+	+	+	+	Beck et al. [12]
12	-	+	No tooth enamel defects or ataxia described	+ Mild cerebellar and brainstem atrophy	Sommerville et al. [11]
13	+	+ Neck muscle weakness	+	+ Prominent cerebellar foliae	Bhatia et al. [13]
14	+	-	Ataxia not described	Not performed	Khamirani et al. [14]

## Discussion

With this systematic review, we present evidence of five reports with 14 relatively homogeneous cases with a mutation in the CTBP1 gene. An additional study (the study by Bathia et al. [13]) was identified in this review, with a case not included in the clinical description by Khamirani et al. [14].

The phenotype of most cases includes developmental and language delay, intellectual disability, motor disturbance, muscle weakness, hypotonia, and cerebellar signs such as ataxia and dysarthria mainly, in addition to dental abnormalities and evidence of cerebellar and vermix atrophy. In some cases, cognitive, motor, and language regression were reported. A case of neurodegeneration and death at 16 years old.

The most-reported mutation (p.Arg342Trp) has been considered a recurrent mutation. Moreover, according to Kaplanis et al. [19], factors associated with recurrent mutations may be attributable to a verifiable phenotype in disease-causing mutations, which makes it easy to identify and report them. Another cause may also be increased mutability at the specific sites, and, finally, positive selection of mutations by “paternal age effect” and clonally expand over time [20]. Determining which factor influences more should be important for future studies. No reports mentioned the age of the parents; for example, developmental disorders caused by de novo mutations have been estimated to have an average prevalence at birth between 1 in 213 and 1 in 448, depending on the parents’ age [21].

Most of the individuals presented de novo mutation. This is quite common, mainly in rare diseases associated with neurological and psychiatric disorders such as intellectual disability, autism, and schizophrenia [22]. In case #14, the authors report the case as de novo mutation [14]. However, the parents are consanguineous, and a brother of the proband affected with a similar condition but not included in this analysis. Although parents were negative for the variant, this would indicate that it could be another mutation in another additional gene causing the disease. It was estimated that people with other affected family members were less likely to have de novo pathogenic mutations [21]. However, in the same study, it has been estimated that approximately 6% of individuals from consanguineous families have a probably pathogenic de novo mutation, which highlights the relevance of considering de novo causality in all families [21].

Of the cases reviewed here, 71% were male. A higher prevalence of autism spectrum disorder, DI, and attention deficit hyperactivity disorder have been observed in males [23, 24]. However, it has been found that women carry more pathogenic variants for brain development than men [25], and it has been observed that males have a 25% lower probability of being carriers of a probably pathogenic de novo mutation compared to females (OR = 0.75, 0.65–0.87 CI 95%) [21]. Thus, it has been considered a gender bias underlying phenotype or social bias [25].

Although reported cases represent highly penetrant alleles associated with single-gene disorders, mutations affecting domains important for protein interactions may also have subtle effects. Only heterozygous variants are found in this review. An autosomal dominant inheritance pattern would be present in family cases, with variable penetrance. CtBPs are coactivators or corepressors of transcription through interaction with other transcription factors and chromatin-modifying enzymes. Therefore, they are unable to bind to DNA independently. A proposed mechanism to explain Mendelian dominance in transcription factors is through a competitive binding [26]. There is competition between the transcription factor allelic variants for binding to the promoter sites they regulate. Nevertheless, this mechanism does not seem to apply to coregulators as CTBP family members.

Oligomerization is a critical factor for transcriptional activity in CTBPs, forming structures in dimers or tetramers by binding to the NAD(H) domain. These molecular complexes promote stability and interactions with DNA-binding factors [27]. Regarding the mechanism of CTBP1 mutation p.R342W to produce disease, a dominant negative effect has been proposed [12]*.* The complexes formed would be a mixture of mutated and wild-type subunits. The dominant negative effect would be more significant when more repeating subunits are included because the mutated subunits block the function of the wild-type molecules [28]. Other mechanisms could be additionally influencing. Mutations can perturb simply protein interactions, as shown by Beck et al. [12]. Another mechanism is stoichiometric imbalances when a certain amount of monomer increases in the complex [28].

We found no published papers with sequence variants in the *CTBP2* gene. The cause of the absence of publications may be due to reduced penetrance and lethality of the mutation with increased prenatal or perinatal death (due to spontaneous abortion, termination of pregnancy due to a fetal anomaly, fetal death, or early neonatal death) [20]. *CTBP2* homozygous null mice die early with brain malformations and axial truncations. This protein is necessary very early in development for exit from pluripotency and the formation of the three germinal layers of the embryonic stem cell [29]. *CTBP2* has unique functions, but many other functions are shared with *CTBP1* [7]. In addition, the *CTBP2* isoform called RIBEYE has different functions in specialized neurons [30]. Variants in exons shared by both isoforms *CTBP2/RIBEYE* could be phenotypically masked and undetected [1].

The possible disease mechanism for CTBP1 mutation p.R342W seems still unclear despite functional evidence of the unstable association of several transcriptional regulatory proteins with the PXDLS-binding cleft, differences in the expression patterns of other genes involved in cellular pathways, and increased pro-apoptotic protein in fibroblasts from patients. The authors have hypothesized an alteration in neurodevelopment due to the absence of apoptotic regulation at the cerebellum level. Animal models with the variant could perhaps give new information. Furthermore, family genetic studies of inherited mutations could help to understand better these two fascinating transcription factors, the relationship between them, and the clinical implications associated with the interaction with their multiple partners.
